# Understanding significant processes during work environment interventions to alleviate time pressure and associated sick leave of home care workers – a case study

**DOI:** 10.1186/1472-6963-13-477

**Published:** 2013-11-15

**Authors:** Gunn Robstad Andersen, Rolf H Westgaard

**Affiliations:** 1 Department of Industrial Economics and Technology Management, Norwegian University of Science and Technology, Alfred Getz veg 3, SB1, 12.etg, N-7491 Trondheim, Norway

**Keywords:** Intervention studies, Health care services, Home care services, Rationalization, Organizational change, Work environment, Ergonomics

## Abstract

**Background:**

Ergonomic and work stress interventions rarely show long-term positive effect. The municipality participating in this study received orders from the Norwegian Labour Inspectorate due to an identified unhealthy level of time pressure, and responded by effectuating several work environment interventions. The study aim is to identify critical factors in the interaction between work environment interventions and independent rationalization measures in order to understand a potential negative interfering effect from concurrent rationalizations on a comprehensive work environment intervention.

**Methods:**

The study, using a historic prospective mixed-method design, comprised 6 home care units in a municipality in Norway (138 respondents, response rate 76.2%; 17 informants). The study included quantitative estimations, register data of sick leave, a time line of significant events and changes, and qualitative descriptions of employee appraisals of their work situation gathered through semi-structured interviews and open survey responses.

**Results:**

The work environment interventions were in general regarded as positive by the home care workers. However, all units were simultaneously subjected to substantial contextual instability, involving new work programs, new technology, restructurings, unit mergers, and management replacements, perceived by the home care workers to be major sources of stress. Findings suggest that concurrent changes induced through rationalization resulted in negative exposure effects that negated positive work environment intervention effects, causing an overall deteriorated work situation for the home care workers.

**Conclusions:**

Establishment and active utilization of communication channels from workers to managers are recommended in order to increase awareness of putative harmful and interruptive effects of rationalization measures.

## Background

Workplace interventions to reduce musculoskeletal complaints rarely achieve their stated objectives or to the extent harmful work exposures are alleviated, such gain tends to be nullified in the longer term. Westgaard and Winkel [1] summarized 59 systematic reviews of intervention studies within this subject area, covering engineering and organizational interventions, interventions aimed at strengthening individual resilience, and reviews considering implementation strategy. The overriding finding was lack of evidence for positive effect on health or risk factors in the longer term. This state of affairs has been noted by many researchers and is increasingly ascribed to concurrent “natural changes”, i.e. on-going changes to workplace conditions unrelated to the intended intervention, yet potentially affecting the intervention outcome and thus considered “noise” when assessing work environment intervention effects [2-8]. Consequently, researchers strongly stress the need for process evaluation as an integral part of intervention evaluation studies [7,9-11], and for coincident changes to be integrated into intervention designs [5].

A general aim and thus a key intervention in both public and private enterprises is a continuous effort to achieve reduced costs and improved quality of their output, whether material goods or services. Such production system interventions or rationalizations (term used in this paper) have a dominant influence on the design of organizations, production lines and workplaces, and are frequently carried out with low priority for worker health effects. Westgaard and Winkel [1] reviewed studies of rationalizations without a stated concern for worker health effects, but nevertheless reporting work exposure or worker health data. Dominant negative exposure and health effects of rationalizations were reported; however, negative effects were to some extent alleviated by management procedures involving a resonant management style [12], worker participation in the design of new production systems and in the rationalization process, organizational support, and procedural justice. The authors posited that on-going rationalizations were a major cause of poor outcome of worker health interventions, and recommended that work environment concerns should be integral to the planning of rationalization efforts, which is also the theoretical basis of the present study. However, few studies provide specific information on the interaction between work environment interventions and rationalization, with equal weight on documenting both processes. The present study aims to contribute to such insight by documenting outcome and processes in a setting where interventions for improved work environment are carried out in an organization that simultaneously strive for production system efficiencies in response to economic constraints.

This study is based on home care workers (HCWs) in a large municipality in Norway. Like most enterprises, the home care services (HCS) has become an object of rationalization measures [13,14]. Many studies report that this occupational group is exposed to several risk factors at work and have health problems. Psychosocial work exposures (e.g. time pressure [15-17], workload pressures [17], high levels of mental job demands [18]) and physical work exposures (e.g. poor ergonomic conditions [15,16,19]) have been identified as risk factors for the prevalent occurrence of musculoskeletal pain for HCWs [19,20]. This also applies to the participants in this study [21]. In 2003 the municipality was served with a legally binding order by the Norwegian Labour Inspectorate (NLI) to improve working conditions due to a high level of unhealthy time pressure. The subsequent work environment interventions were duly carried out by the municipality. At the same time, the HCS underwent several organizational changes and other significant processes influencing work duties took place. The stated ambition of the municipality was that the work environment interventions would improve risk factors of time pressure and ergonomics, and further reduce sick leave. However, musculoskeletal symptoms and sick leave remained high at the end of a 6-year observation period [21]. The present study, using a historic prospective mixed-method design, documents sick leave development, here used as a work environment and health indicator, rationalization measures and work environment interventions, and their effects as perceived by the HCWs.

The specific aim of the study is to identify critical factors in the interaction between the two processes, whereby putative positive work environment intervention effects are reduced or eliminated by rationalization. The study setting is well suited to explore this point: The NLI inspection and subsequent orders ensure that an intervention for improved work environment has high legitimacy, and key stakeholders in the municipality showed a genuine interest in achieving good work conditions for HCWs. Furthermore, the HCS has been subjected to organizational changes to reduce costs, obtain more efficiency and provide improved access to services, like most health care systems in the industrial world [22-26].

## Methods

The study has a mixed-method design by using a combination of quantitative estimations (of perceived time pressure and evaluation of intervention effect), register data of sick leave, documentation of significant events and changes, and qualitative descriptions of employee appraisals of their work situation. The study is part of a larger longitudinal study of factors contributing to an undesirable quality of work environment and sick leave rate in the HCS.

### Setting and case description: The HCS Campaign and subsequent Interventions

In 2002–2008, the NLI carried out a national campaign focusing on the work environment in the HCS. The purpose of the campaign was to target occupational risk factors identified by a national survey to be highly prevalent among HCWs: time pressure (characterized as straining by 80% of respondents), ergonomics and violence/threats [27], and stimulate to actions that reduce such risk factors. In 2003 the municipality in this study received orders from NLI due to a high level of unhealthy time pressure and a high sick leave rate. The municipality was legally obliged to comply with NLI orders, and responded by allocating NOK 14.5 mill (€2 mill). The anticipated result of their interventions was reduced time pressure and lower sick leave of HCWs.

A project group with worker participation was established, and a model for risk assessment was developed, comprising ten potential risk factors of time pressure (e.g. work organization, patient characteristics, resources available, management, culture etc.) anticipated to result in physical and mental complaints and furthermore sick leave. The model served as basis for a thorough work environment survey involving all HCWs. A program focusing on employee empowerment, skill upgrading and networking was established as a process method to ensure worker participation. Employees at all units provided descriptions and examples on risk factors of time pressure, and participated in defining intervention content. Contributions were listed on flip-overs, further discussed and then written in a formal document stating the identified risk factors, associated interventions, person in charge of each particular intervention, due date and so on. Internal reports point at work organization, patient characteristics, and lack of resources as the risk factors mentioned most frequently across the units (not referred to due to anonymity). In 2004–2006 interventions were carried out in local units to target unit-specific risk factors of time pressure, and on municipal level to target common risk factors for all units. Examples of local interventions included the establishment of a functional template for work lists, clarification of appropriate expectations by patients, and buying more cars and telephones. Common interventions for all units included the implementation of a safety patrol to relieve stress due to alarms going off, a temporary staff recruitment service to fill vacancies due to high sick leave, and the introduction of staff uniforms to advance HCW professionalism. Also, the campaign and its corresponding actions have generally motivated the municipality to ensure a continuous focus on work environment issues for this professional group.

In 2008 NLI was pleased with the municipality’s efforts and closed the orders, implying that the work situation would be improved for the targeted risk factors. In their campaign evaluation report the NLI referred to this municipality as an example of ‘good practice’ in responding to orders given. However, HCWs in 2009 still seemed exposed to several occupational risk factors, musculoskeletal health complaints, and high sick leave rate [21].

### Participants

At study start, the HCS of the municipality was organized in 11 geographically separate home care units. Representatives of the municipal secretariat informed the unit leaders about the project and 6 units signed up for participation. HCWs with employment fraction ≥ 50% (181 participants) were included in the study. The final sample consisted of 138 respondents (76.2% response rate to the questionnaire), of whom 89.8% were female. 77.5% had a professional health care education as either Registered Nurse or Enrolled Nurse. Average age was 42 years (range 20–64). Seventeen HCWs were selected as interview informants through purposive sampling based on seniority (minimum 7 years) and employment fraction (≥50%).

### Procedure

Initial conversations were carried out with an inspector of the NLI, representatives of the municipal secretariat and unit leaders to gain insight in the NLI campaign, and aspects of the HCS including the organization of work duties, organization-specific work demands and significant changes and events relevant for the composition of the questionnaire and interview guide. Prior to the data collection, one of the researchers participated on staff meetings at each unit to present the study and give practical information about participation. Questionnaires in paper format were put in an envelope together with a letter of information and an informed consent form, and placed in each employee’s personal shelf at work. An inquiry concerning interview participation was placed in selected personal shelves. Filled-in questionnaire and informed consent form were to be returned in a provisional sealed mail box placed in the staff room within two weeks. Two reminders were sent by letter to increase participation. Respondents and informants were remunerated with NOK 200 (=27€) and NOK 300 (=41€), respectively. The data collection was carried out between March 26 2009 and June 17 2009, and was finally closed on June 29 2009. The study was approved by the Municipal Executive, the Regional Committees for Medical and Health Research Ethics (REC) (no. 4.2009.19) and Norwegian Social Science Data Services (NSD) (no. 21036).

### Data collection

The questionnaire comprised altogether 129 items. The present study utilizes self-formulated items regarding perceived changes in working conditions the last 5 years. The respondents were asked to compare the present situation to the past with regards to perceived time pressure. Response categories ranged from 1 (considerable less) to 5 (considerable more) with a neutral mid-point, recoded to a three-point response scale (“less”, “no change” and “more”). The respondents were also asked to evaluate the success of local work environment interventions with four response alternatives; ‘a failure’, ‘less good’ , ‘quite good’ , ‘a success’ , recoded to a dichotomous variable; “no effect” and “positive effect”. IBM Statistical Package for Social Sciences (SPSS) version 19 was used to compute frequency distributions. Finally, open-ended questions on significant positive and challenging changes affecting the work situation where respondents could submit self-formulated responses were also included.

Seventeen semi-structured, in-depth interviews were carried out with Registered Nurses and Enrolled Nurses. The interviews lasted approximately 1 hour and were audio-recorded and later transcribed verbatim. The interview guide was based on initial conversations with unit leaders and municipal representatives, and covered topics concerning work environment, work tasks, and perceived changes in such the last years. The paramount questions were “How do you perceive your work situation today?” and “How do you perceive changes in your work situation to affect you and your work?” Main questions were followed by probe questions such as “Can you give an example of this?”, “What do you think caused this?” and so on to stimulate rich descriptions.

Annual sick leave statistics from 2004–2009 for each unit were accessed from the municipality’s records, and contextual information regarding significant events and changes taken place the last 7 years was gathered through interviews with representatives of the municipal secretariat and unit leaders.

### Qualitative analysis

The open responses in the returned questionnaires were organized to identify topics/categories related to perceived changes in the work situation. As the majority of these responses were briefly formulated, organization and categorization was straightforward. The interview data were analysed by Template Analysis [28,29] producing a hierarchical list of codes representing themes identified in the interviews. The software QSR NVivo 9 [30] was utilized to aid in organizing and examining the data. The interview guide served as basis for an initial template consisting of three higher-order themes: 1) “Appraisal of work situation” with the sub-themes “sources of stress” and “sources of job satisfaction”; 2) “Changes affecting the work situation” with the sub-themes “organizational changes”, “work environment interventions” and “production system rationalization”, and finally 3) “Consequences” with the sub-themes “individual level” and “group- and organizational level”. The analysing process of the interview data was carried out by identifying higher-order themes and further scrutinizing the contents of these themes to identify and differentiate lower-order themes. Accordingly, the initial template was somewhat modified throughout the analysing process, resulting in a final template presented in the Results section. This final template served as basis for interpretation and illumination of the data, in line with recommendations by King [28].

## Results

**Figure 1:**
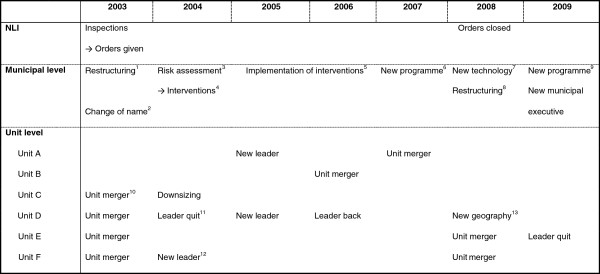
**A chronological timeline of significant events taking place in the municipality from 2003 to 2009.** Note: ^1^Separating home help (practical tasks) from home care resulting in pure professional caring tasks. ^2^Change of name from “Health and care services” to “Health and welfare services”. ^3^Risk assessment resulting in ^4^interventions as described in Method section. ^5^Implementation of interventions on municipal level affecting all units included the establishment of a safety patrol, temporary staff recruitment service and staff uniforms. Implementation at unit level involved improved work routines and organization of work, more equipment and facilities etc. ^6^Quality-enhancing work programme involving specification of new work duties and responsibilities concerning the everyday life of patients living at home. ^7^Introduction of a Personal Digital Assistant involving changes in work procedures, acquiring of new skills etc. ^8^Assisted living institutions separated from the Home Care Services resulting in pure *home* care. ^9^Quality-enhancing work programme involving specification of new work duties and responsibilities concerning patients’ discharge from hospital and return to home. ^10^Unit mergers in all cases involved new work office, leader, colleagues, geographical area, care recipient group, budget figures, work routines, organizational culture etc. ^11^Leader quitting in all cases involved a turbulent period of stand-in leaders before the hiring of ^12^new leader. ^13^New geography involved new patients and greater geographical distances of transferring.

Figure 1 shows a chronological summary of significant events for the HCWs in the municipality from 2003 to 2009. Campaign-related actions are reflected on NLI and municipal levels, yet separate events initiated on municipal level with consequences for local units are reflected on both municipal and unit levels. Examples involve mergers for all units (some repeatedly), changes to middle management and executive management, organizational changes such as the introduction of new technology, new work programs, and restructuring by separating the home *help* service and assisted living institutions from the HCS (i.e. HCWs should no longer attend to non-medical needs or to care recipients living in institutions). All of these changes impact on work tasks, work duties and workday organization of the HCWs.

**Figure 2:**
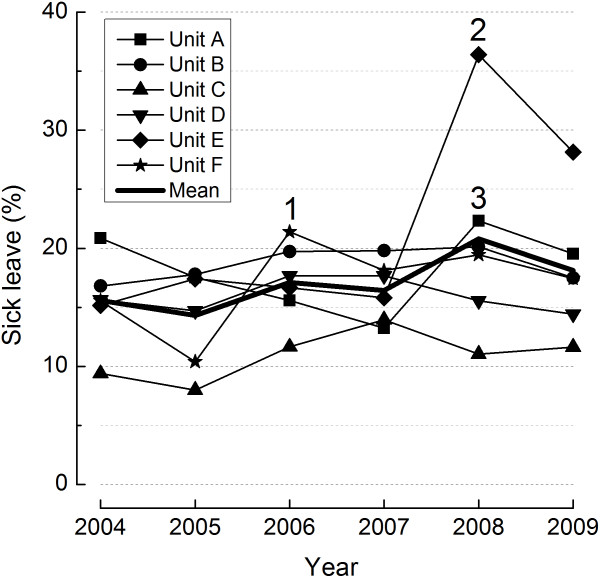
**Sick leave statistics for each unit and for the total sample of home care units (A-F).** Note: Marked peaks in sick leave are labelled: 1. unit F (2006) sick leave peak with no clear coincident event; 2. unit E (2008) sick leave peak coincides with merger; 3. unit A (2008) sick leave peak with no clear coincident event, yet qualitative findings indicate difficulties in the aftermath of merger.

Sick leave statistics for each unit and the total sample are shown in Figure 2. Statistics from 2003 are not included as rates are incompatible due to the implementation of a new basis for statistics, and a marked reorganization of the home care units (from 21 to 11 units). A general tendency of increasing sick leave is observed, with detectable differences between the units. Marked inflections in the sick leave of units are labelled and commented in the figure legend. Clear, sustained differences in sick leave between units were noted and attempted understood in interviews, yet no firm explanation of these differences emerged.

**Figure 3:**
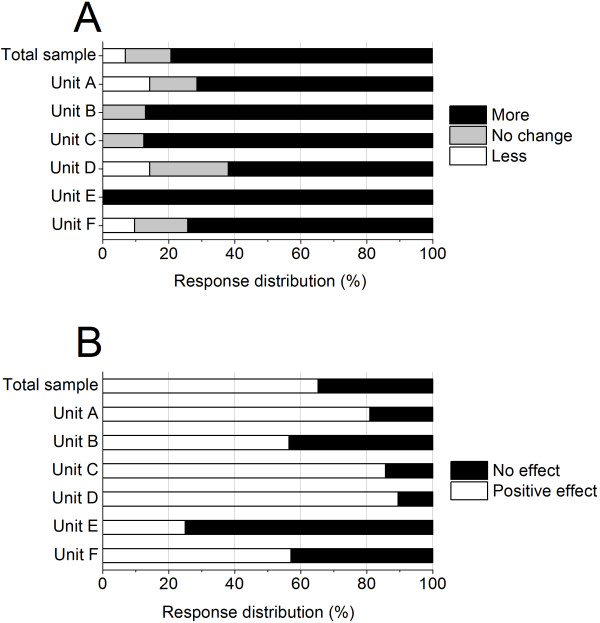
Frequency distribution of (A) perceived change in time pressure over the last 5 years, and (B) evaluation of intervention effect, for the total sample and for the individual units.

A large majority (79.2%) of the HCWs perceived an increase in time pressure over the last 5 years, with unit assessments varying from 100% (unit E) to 61.9% (unit D) (Figure 3A). Further, 65.3% of the HCWs considered the work environment interventions to have had a positive effect in improving their work situation. This evaluation also varied among the units, ranging from a high 89.5% positive (unit D) to a low 25% positive (unit E) (Figure 3B).

By inspecting Figures 2 and 3, some units stood out by more positive assessment of the work environment interventions (units A, C, D) and also recorded lower sick leave (units C, D). Others perceived more time pressure (unit E), recorded high sick leave (unit E), and less positive intervention effects (units B, E, F).

82 HCWs responded to the open questions on changes that have affected their work situation in positive (n = 55) and challenging (n = 70) directions. Many of them listed several changes, for a total of 178 comments. Comments concerning positive changes were intervention-related, emphasizing improved organization and more available equipment and facilities. Comments identifying challenging changes were closely intertwined and dealt with categories related to increased exposure (time pressure and workload), implicit health consequences of increased exposure (high sick leave), as well as perceived causes of increased exposure (organizational changes, large units, budget cut-backs and tighter time allocations). As these categories were closely connected to the qualitative interview findings presented below, they were merged into Table 1 by showing number of open comments in the questionnaire corresponding to template themes.

**Table 1:** Final coding template of interview data with quotes, examples of descriptions and numbers of responses to open survey questions corresponding with interview data

**Final template representing themes**	**Quotes and examples of descriptions**
**1. Strenuous work situation**	
1.1 Time pressure (21)	*“I know several colleagues who look for other jobs, because they are – it is the time pressure that wears us down, terribly. Yeah, it is tearing on us, it is the worst part, the absolute worst”*
1.1.1 Physical strain	*‘being highly strung’; ‘working at a high pace’; ‘running from place to place’*
1.1.2 Mental strain	*‘being preoccupied with the next task on the work list’; ‘worrying about not having time to complete all tasks and getting to the next patient in time’*
1.1.3 Emotional strain	*‘feeling bad about not having the time to do the job properly’; ‘having to leave patients behind’; ‘feeling drained by constantly being behind schedule’*
1.2 Organizational demands	
1.2.1 Full work lists	*‘five visits are listed at 09. a.m.’; ‘no time for lunch’*
1.2.2 Patient characteristics	*‘more demanding diagnosis’; ‘more complex cases’*
1.2.3 Distribution of work lists (6)	*‘extra assignments handwritten on my work list’*
1.2.4 Unexpected incidents	*‘finding a dead patient’;’someone requiring extraordinary care’*
1.2.5 Indirect time (5)	*‘Time for transferring is not included, and I move around by car for maybe 40–50 minutes – before 12 a.m.’*
1.3 Conflicting work demands	*“Time pressure makes you mentally drained because you’re not able to do what you are supposed to […]. Comforting, talking, stroking their backs, for example, make them breakfast on a Sunday morning. The caring part of the job is disappearing.”*
**2. Changes affecting the work situation**	
2.1 Increased workload (19)	*“Well, we have all these requirements directed at us, a lot more now than it used to be, about documentation and all kinds of stuff we must register and – we’re not able to do half of what we are supposed to…”*
2.1.1 Additional work tasks	*‘We get more and more tasks’; “We get more and more patients but not more staff, right”*
2.1.2 Efficiency demands (9)	*‘They* [municipal level] *are very eager at cutting down and cutting down time’. ‘Some years ago, no visits were less than 15 minutes, now they’re down to 5 minutes’.*
2.2 Unit mergers (17)	*“There have been some organizational changes, you know, and it tears on us – When they start with all that, I’m just like: AGAIN!?”*
2.2.1 Strain related to the process	*‘lack of information and participation’;’employee resistance and dissatisfaction’*
2.2.2 Strain related to the consequences (16)	*‘culture clashes’; ‘unit size too large’; ‘chaos’; ‘larger geographical distances’; ‘new localities’; ‘establishing and mastering new roles’; ‘new ways of cooperating’*
2.3 Budgetary constraints (10)	*“You are told that the budget situation is getting worse and worse. And you are told that you have to do more and more in less time. It affects you, you feel; Ok, fine, there is a limit for – yeah – for what you can handle”*
2.4 Work environment interventions	*“We have done a lot of work… Routines are in place, the work is better organized. The unit is run much, much better”* versus *“It fails after a while anyway. When the council’s economy is poor, we go back to where we used to be”.*
2.4.1 Improved organization (30)	*‘work lists are organized based on geography and a steady group of patients’*
2.4.2 More equipment and facilities (28)	*‘we have more equipment now’; ‘more cars and telephones’*
2.4.3 Improved routines for cooperation (7)	*‘relieve each other’; ‘call each other if someone has time available’*
**3. Consequences of strenuous work situation**	
3.1 Worker health	*“I get pain in the neck, shoulders and head, get stiff-necked, and it is not just me – many of us struggle with our shoulders, neck and head. It is the time pressure that tears on all of us, that kind of stress, you know.”*
3.2 Job performance	*‘indirect tasks are postponed’; ‘leaving parts of the job undone’;* ‘*being forced to prioritize’*
3.3 Sick leave (10)	*“I remember… Earlier, if someone called in sick, we never talked about it. We just said ‘Oh! Get well soon!’, but today we’re more like ‘Oh my God, TODAY as well!’.”*
3.4 Work environment	*“Now the unit is a lot less clear, there are too many people to deal with. […] There is more commotion and noise; I feel it is hectic because of all the people around you”; ‘we get irritable when we’re stressed’*

Note: Full quotes are illustrated by double quotation marks (“), whereas partial quotes (i.e. a meaningful description disconnected from a full sentence) are illustrated by single quotation marks (‘). Numbers in parenthesis indicate number of responses to open questions in questionnaire corresponding to themes emerged from the interviews.

### Qualitative research findings

Table 1 shows the final template, presenting interview findings in terms of higher-order themes and lower-order themes illustrated by quotes and examples of descriptions presented by the HCWs. Three higher-order themes emerged in the data: 1) strenuous work situation, 2) changes affecting the work situation, and 3) consequences of strenuous work situation, with several lower-order themes developed within each of them.

### Strenuous work situation

Several distinct sources of a strenuous work situation emerged from the interviews. All of the informants spontaneously described their work day as busy, hectic, stressful, and characterized by a constant fight against time. Time pressure was generally considered to be the most strenuous work factor, manifesting itself as physical, mental and emotional strain. Several informants pointed at a negative trend towards increasing time pressure, consistent with the assessment presented in Figure 3(A). Five themes relating to organizational demands (“placeholder code”) perceived to cause a strenuous work situation emerged, all considered to result in work overload and time pressure. Work lists consisting of descriptions of work tasks and visits to be carried out, including specified time estimates, were described as exceeding realistic expectations. Several specified tasks/visits were listed as to be carried out simultaneously, and tight time allocations allowed no tolerance for extraordinary incidents. Patient characteristics were described as becoming more demanding as patients were sent home from hospital at an earlier stage of recovery. Some patient groups (e.g. drug abusers and psychiatric patients) needed time that exceeded the standardized time allocation, disarranging the time-specified work lists. Distribution of work lists containing unexpected elements (e.g. new patients or new geographical areas), which occurred due to tight budgets and restricted hiring of stand-ins, further induced work overload and delays. Unexpected incidents were described as happening quite frequently, without the system taking such incidents into account. Activities indirectly related to patient care were described as an increasing source of work strain. These were not specified tasks in the work lists and thus perceived not to be covered by allotted resources. Overall, such organizational demands led to an increasing strenuous work situation for the HCWs.

The informants further described strain due to conflicting work demands, in particular the conflict between internal and external demands. They expressed clear self-directed expectations of how the job should be performed, often involving compassionate activities beyond what was stated in the individual agreement concerning what medical help the patient is entitled to receive. These expectations were perceived to be in contrast to requirements directed from municipal level focusing on rapidly carrying out professional nursing activities at the expense of caring activities.

### Changes affecting the work situation

When asked to compare the present work conditions to the situation 5–6 years ago, all informants described a negative trend of increased workload, counter-productive organizational changes and budgetary constraints. A fourth theme, work environment interventions, was described in positive terms by some of the informants, but this topic had to be probed to generate a response and descriptions were two-edged. All informants described a tendency towards more challenging work situation characterized by increased workload and higher work pressure with less time available. The further elaboration of causes for this trend coincided with descriptions already put forth, such as more demands of indirect time activities. Higher efficiency demands were described in terms of work tasks being more specified and standardized, and time allocated to specific work tasks being reduced.

Informants of all units exposed to merger in the study period (all but unit C) described this change as resulting in a more challenging work situation. Stress-inducing effects were related to both process issues and consequences. Unit mergers were perceived motivated by cost saving and not rooted in concern for patients or employees. Over all, budgetary constraints were considered as the antecedent of several processes resulting in impaired working conditions, and the situation was perceived to get worse every year. Economic deficits, causing restrictions to hiring temporary workers and filling vacant posts, were perceived to generate increased workload and sick leave.

Work environment interventions that describe positive changes emerged as a theme after explicit probes. Improved organization of work lists, additional equipment such as cars and telephones, and improved routines for cooperation were described as effective initiatives in reducing work strain. However, some informants couldn’t think of any specific intervention for improved work environment, and a few HCWs were more pessimistic in their descriptions, explaining how the interventions diverted time and money from the unit, were not followed up due to lack of time, were withdrawn due to lack of resources or the new situation was lapsing back to the former situation.

### Consequences of strenuous work situation

The informants described several destructive consequences of strenuous working conditions and negative changes to the work situation, which thematically could be distinguished as being on individual level or on group- and organizational level. On individual level, the majority of informants described how their health had been impaired due to work-related stress and worries, but also because of wear and tear injuries. Frequent descriptions included exhaustion, tension in neck and shoulders, headaches, back pain, and strain injuries. The informants described reduced job performance with deteriorating service quality as a consequence. On group- and organizational level, the informants described how high sick leave was a twofold problem for the units. It was regarded as a symptom of a strenuous work situation due to substantial pressure and onerous organizational changes, and a source of additional strain on the remaining workers because of work overload. With increasing work pressure due to restricted filling of vacancies, informants described how the atmosphere would get affected when co-workers call in sick. A few informants described how the work environment had improved due to the interventions, yet most informants described negative effects on the work environment in times of stressful peaks. Unit mergers and larger unit sizes were perceived to result in work environment commotion and over-complexity.

## Discussion

The results of this study confirm the initial hypothesis and thereby the assumption implicit in the study aim: on-going rationalization measures interact with work environment interventions and lessen the impact of these, highlighting the multiple processes that determine working conditions of HCWs. The NLI initiated work environment interventions were in general perceived to have a positive effect by improving targeted areas identified to cause unhealthy time pressure and work strain. In parallel, new sources of time pressure that negate the positive effects of work environment interventions were introduced. The result is an overall worsening of the work environment. The documentation of the many processes involved in managing the HCS supplemented by detailed worker descriptions, provides a basis for a better understanding of their overall effects on work environment, and provides a case-based example of how ergonomic and stress interventions can fail.

**Figure 4:**
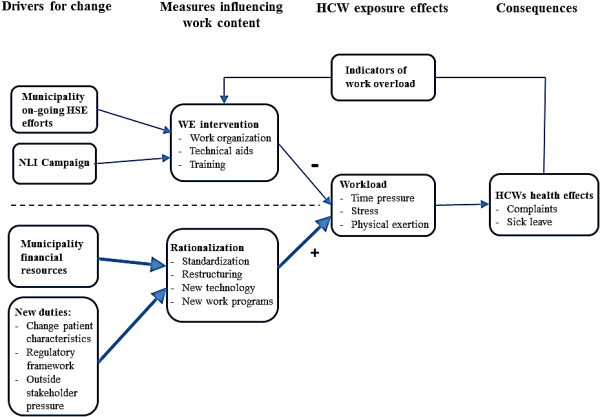
**Graphic summary of results.** Note: Dashed line symbolises a distinction of the two concurrent processes in the organization of the HCS, and a putative lack of consideration and inter-level communication of work environment effects for HCWs. Bold arrows indicate dominant processes. Plus and minus signs on arrows indicate increasing and decreasing effects on workload, respectively.

Figure 4 presents a graphic summary of the results: A number of drivers for change are listed, including the NLI campaign to reduce time pressure, but also powerful drivers unrelated to the work environment efforts of the municipality. These include costs control and incentives to improve service quality. These drivers are omnipresent and have strong and continuous effects on work organization and workload, symbolized by bold-faced arrows. Measures to improve the work environment had positive effects on working conditions of HCWs; as such, the municipal responses to NLI orders were successful. However, the NLI campaign did not address the many changes to working conditions carried out independently, and had the flavour of a one-off effort, supplementing the traditional health and safety activities, but was not properly sustained over the study period. Simultaneous production system rationalization measures had powerful negative consequences on workload, and tipped the balance towards more difficult working conditions. External changes to the organization of the health service with early discharge from hospitals resulted in more demanding patient characteristics, and regulatory framework and outside pressure pushed for improved quality of care. Such demands were met by traditional productivity-enhancing management actions; foremost among these were standardization measures. Consequently, time pressure and work stress were perceived to increase over the study period. The resulting health effects of work strain for this group of HCWs; musculoskeletal complaints, have been documented elsewhere [21]. Finally, a feedback loop is indicated: municipal stakeholders respond to high sick leave and other indicators of work overload by attempting to improve working conditions. Such supplementary work environment measures in general do not relate to work content determinants that result from efficiency measures. The overview in Figure 4 is not unique, several studies propose organizational changes or turbulence unrelated to an intervention as a plausible explanation of lack of positive results [3,6-9] and conversely, that organizational stability is a prerequisite for interventions to succeed [5]. However, the present study aims to demonstrate a more nuanced documentation of critical factors and processes implicit in causing an unfavourable outcome.

Most positive intervention-related changes identified by the HCWs were of a specific character, and were directly related to areas identified as risk factors of unhealthy time pressure, implying that interventions successfully hit targeted areas (e.g. buying more cars, sorting out work lists). However, the overall effect of the work environment interventions seemed small in comparison to powerful effects of the rationalization measures.

Restructuring by separating practical home help from the HCS was part of a process that allowed further standardization of specialized caring work duties. Although intended to reduce work stress by removing ‘disturbing’ tasks such as house cleaning and grocery shopping, it may conversely have led to increased work intensification by reducing work porosity as such practical tasks often were postponed in situations with high time pressure. Workers providing nursing care in other studies report significantly more strain than workers providing personal care [31]. The standardization of work duties allowed work lists to be more precise in describing each work task with corresponding time allocation, making work tasks more efficiently allocated and resulting in more hectic working conditions. More visits of shorter duration also generate more indirect-time demands (transport, documentation), which was not time-compensated: the overall time allocated to caring tasks as specified on work lists was not reduced. This is in accordance with other studies referring to fragmentation of care time and increasing time pressure as the core problems facing HCWs [14]. Standardization measures in this study is an example of a trend towards a Taylorising type of care, operationalized by a specified amount of minutes to fragmented physical work tasks (e.g. putting on support stockings should take 5 minutes), and valuing instrumental care over affective care [32]. HCWs in the present study described how being in a constant hurry prevented them from yielding affectionate care, causing emotional strain and health complaints by feeling unable to yield sufficient care [21]. Into the study period the municipality changed the health division’s name from “Health and *Care* Services” to “Health and *Welfare* Services”, signalling the elimination of caring aspects. Cloutier, David, Ledoux, Bourdouxhe, Gagnon and Ouellet [25] refer to home care personnel experiencing an erosion of job content as the affective aspects of their work are disappearing due to work intensification caused by restructuring. It is hypothesized that the stakeholders who effectuated the standardization process in this study, were not aware or chose to ignore the emotional aspects of HCWs work duties, while they were fully aware of the need for productivity enhancing measures. The interviews further suggest that other, more incidental “time thieves” (e.g. difficult traffic conditions, patients requiring extraordinary care) were not properly recognized in the standardization process. Such items are clearly dependent on context and probably also on the individual HCW, and are difficult to integrate in time plans. Other perturbations include extra patients on work lists due to HCWs calling in sick, a problem with workload implications that may not be fully communicated to municipal administrators.

Documentation of work tasks is necessary in efforts to improve the quality of HCS. However, administrative requirements were listed as a major contributing factor to increased workload. Two new work programs introduced during the study period involved significant increases in documentation needs. Service quality was to be increased by introducing a “memory list” specifically stating what to observe, how to act and what to document in a care recipient’s home. The HCWs experienced that work duties related to these programs resulted in additional work tasks at the expense of traditional caring tasks, and as they mainly involved duties indirectly related to the care recipients and presumably already carried out though in a less systematic manner, no extra time was allocated. Cloutier, David, Ledoux, Bourdouxhe, Gagnon and Ouellet [25] refer to such administrative responsibilities as ‘invisible tasks’ resulting in work intensification as they are added without eliminating traditional tasks. Hence, these two programs most likely introduced additional sources of time pressure for the HCWs. Likewise, the implementation of new technology, a Personal Digital Assistant (PDA) required new skills and new working methods, likely contributing to additional work. One rationale behind the introduction of the PDA was that by having information available at all times and writing reports immediately after an assignment, waiting time for access to a computer would be reduced. The flip side involved a stated intention of increased efficiency as ‘time waiting in line’ (“waste” in rationalization terms) would be reduced.

A rationale for restructuring the organization by separating the assisted living institutions from the HCS and merging pure home care units, was to increase quality by making the services more specialized and to save costs by reducing the need for administrative personnel. Larger units were assumed to be more robust and less sensitive to disturbances such as unexpected sick leave. Organizational change in terms of unit mergers is accepted to be a stressful experience for employees, and the human costs of such mergers is put forward as one explanation as to why so many mergers fail in reaching their stated objectives [33-35]. During the study period, all six units underwent mergers, two of them repeatedly. The HCWs expressed merger-related stress both with the change process itself and with the subsequent consequences: increased time pressure was attributed to larger unit sizes, greater geographical distances, establishment of new roles and ways of cooperating, and culture clashes. These factors remained disruptive elements independent of the work environment interventions. HCWs who worked in small units forced to merge with larger ones, were particularly vulnerable to merger-related stress. For them the merger implied a new work situation involving new office, new colleagues, new leader, new geographical areas with new care recipients, new work routines, new organizational culture, new budget figures etc. Two marked peaks in the sick leave statistics (units A and E) appear to reflect strain upon merger, by correspondence in time and by the qualitative data. Previous research has linked organizational downsizing to sick leave [36], but strong associations have also been found between workplace expansion and sick leave [37].

The discussion has considered factors that may explain the disappointing outcome of the NLI initiated interventions; a relevant question is why the negative development is allowed to happen: a sick leave of nearly 20% is clearly a worry for all parts involved. At one level, it is clear that financial and quality considerations are given priority, while workload consequences of rationalization interventions may not be fully understood. Findings suggest that stakeholders on higher organizational levels do not have intimate insight in work demands on HCWs, and that commonly used work descriptors may differ in content by organizational level (manuscript in preparation). The responsibility for good working conditions and budget managing reside with the unit leaders, and may involve conflicting concerns. Municipal staff being two organizational levels removed (unit leader being the intermediate level), may be too distant from the ‘shop floor’ to manage the integration of work environment concerns with the concern for effective production of high-quality services. Disruptions to work duties due to organizational restructuring may not be fully understood. Better insight in the interruptive influences of rationalizations would be beneficial to stakeholders; such insight may exist at the intellectual level, but is not necessarily internalized so that it can be incorporated when planning changes to the production system. Improvement in two-way flow of information and inclusion of HCWs in the planning and implementation of production system rationalizations seem a key ingredient in preserving good work environment while accommodating (necessary) productivity-enhancing measures.

Some units seem to benefit more from work environment interventions while others suffer more from rationalizations. It has not been possible to identify trustworthy explanations of between-unit differences in responses. However, differences in the way unit leaders have dealt with change processes (e.g. information flow, employee participation, handling employee reactions) and change consequences (organization of enlarged units, culture clashes, training) have likely affected perceived exposure effects and subsequent consequences [1], and may explain such variations. Leader stability and the implementation of interventions likely varied across the units. Other between-unit factors include geographical extension (impacting on transferring distances) and patient characteristics (psychiatric diagnosis and drug addiction more densely populated in certain areas), both involving work time exceeding allocations. Finally, considerable unit variations in excess spending and budget deficit caused variations concerning practice of hiring temporary staff when sick leave, and may explain variations in perceived overload and time pressure. Sustained differences in sick leave between units imply a potential for developing ‘best practice’ management at unit level, an opportunity that should be explored.

The use of mixed-methods design in this study is in accordance with recent methodological recommendations revolving around the need for a more eclectic and complementary approach in order to understand the process issues in intervention research [5,9,11,38]. In the present study, a comparison of sick leave rates or of quantitative measures pre and post intervention without including qualitative interviews, open-ended survey questions and the documentation of concurrent changes would have yielded insufficient and incorrect results regarding intervention effect. Programme or theory failure and implementation failure [9] are common suggestions to disappointing intervention results and could have been wrongly set forth as explanatory factors. Also, the units have changed to such a degree that a comparison would not have been meaningful. Turnover or changes in staff compositions may pose potential problems when comparing sick leave development within and between units. Changes to the extent demonstrated in this study are the rule rather than the exception in today’s working life. Generalizability of the present results is limited due to the nature of case studies. However, general principles regarding the interference of concurrent changes during interventions as posited in Figure 4 appear valid for most organizations and can be transferred to branches outside health care. Whereas most studies mention the interfering role of concurrent changes in an anecdotal manner, this study adds to the existing literature by systematically examining significant changes and events over a 7 year period, and elaborates on these elements by including worker perspective through qualitative methods. Open-ended survey responses and interview descriptions were strongly accordant, strengthening the trustworthiness of the findings. A historic prospective design may involve certain pitfalls such as memory bias or an overestimation of informants’ abilities to reflect on the impact of past incidents. However, this sense-making process is necessarily a retrospective activity [39].

## Conclusions

Concurrent production system rationalization measures resulted in negative work exposure effects that negated positive effects of a comprehensive work environment intervention program carried out in the HCS in a Norwegian municipality. Substantial contextual instability occurred during the intervention period, such as new work programs and several organizational changes with implications for work duties, work content and workload. As change and restructuring for improved performance are inevitable parts of organizations’ lives, it is necessary to be aware of work environment consequences of such activities. It is recommended to establish and actively utilize communication channels from workers to middle and top-level management to increase awareness of putative harmful effects of rationalization measures.

## Competing interests

The authors have no conflicts of interest or competing financial interest to declare.

## Authors’ contributions

GRA participated in the design of the study, the collection of data, analysis and interpretation of data, and drafting of manuscript. RHW participated in the design of the study, interpretation of findings, and drafting of manuscript. Both authors read and approved the final manuscript.

## Pre-publication history

The pre-publication history for this paper can be accessed here:

http://www.biomedcentral.com/1472-6963/13/477/prepub
